# An NGS-based assay for accurate detection and quantification of immune gene expression in mouse tumor models

**DOI:** 10.1371/journal.pone.0303171

**Published:** 2024-05-20

**Authors:** Jia Xue, Xiaobo Chen, Xiaoyu An, Jingjing Wang, Mingfa Zang, Binchen Mao, Sheng Guo, Tao Yang, Rajendra Kumari, Qi-Xiang Li

**Affiliations:** Crown Bioscience, Inc., San Diego, California, United States of America; Pennsylvania State University Hershey Medical Center, UNITED STATES

## Abstract

Tumor microenvironment (TME) is a complex dynamic system with many tumor-interacting components including tumor-infiltrating leukocytes (TILs), cancer associated fibroblasts, blood vessels, and other stromal constituents. It intrinsically affects tumor development and pharmacology of oncology therapeutics, particularly immune-oncology (IO) treatments. Accurate measurement of TME is therefore of great importance for understanding the tumor immunity, identifying IO treatment mechanisms, developing predictive biomarkers, and ultimately, improving the treatment of cancer. Here, we introduce a mouse-IO NGS-based (NGSmIO) assay for accurately detecting and quantifying the mRNA expression of 1080 TME related genes in mouse tumor models. The NGSmIO panel was shown to be superior to the commonly used microarray approach by hosting 300 more relevant genes to better characterize various lineage of immune cells, exhibits improved mRNA and protein expression correlation to flow cytometry, shows stronger correlation with mRNA expression than RNAseq with 10x higher sequencing depth, and demonstrates higher sensitivity in measuring low-expressed genes. We describe two studies; firstly, detecting the pharmacodynamic change of interferon-γ expression levels upon anti-PD-1: anti-CD4 combination treatment in MC38 and Hepa 1–6 tumors; and secondly, benchmarking baseline TILs in 14 syngeneic tumors using transcript level expression of lineage specific genes, which demonstrate effective and robust applications of the NGSmIO panel.

## Introduction

Immunological components not only play vital roles in the pathogenesis of many human diseases, including cancers, but also in the disease pharmacology. Modern tumor immunology reveals that tumor microenvironment (TME), including tumor-infiltrating leukocytes (TILs), cancer associated fibroblasts (CAF) and other tumor stromal components (*e*.*g*., endothelia), all play fundamental and complex roles in tumor development and pharmacology, particularly the pharmacology of immuno-oncology (IO) treatment [1–4]. Cancers are heterogeneous diseases, not only reflected in tumor cell genetics but also in TMEs. We recently demonstrated that intrinsic tumor immunity of four tumor models (MC38, Hepa 1–6, CT26 and EMT-6) determines their responses to immune-checkpoint inhibitor (ICI) [3]. Therefore, a comprehensive understanding of the tumor immunity is critical for revealing mechanism of action (MOA) and predictive biomarkers of IO treatments [5].

Multi-parametric (including multi-omics) profiling of various tumors is important to reveal baseline tumor immunity as well as their corresponding pharmacodynamics. For instance, multi-color flow cytometry analysis is the most common method to reveal TILs in bulk tumor tissues [5], while whole transcriptome sequencing (or RNAseq) [3, 6, 7] and microarray [8–10] analysis can reveal immuno-genomics, and proteomics can expose immune-proteomics parameters of tumors [3]. Pathology-based approaches, *e*.*g*., immunohistochemistry (IHC) and immunofluorescence (IF) can also be particularly revealing for the spatial distribution of the immune-components in tumor tissues. Apparently, different methodologies have pros and cons in their applications, so one should carefully consider methods according to the study objectives.

Mouse tumor models, including syngeneic cell derived tumors and mouse tumor homografts [4, 5], along with various humanized mouse tumor models [11], are the main workhorse of IO research, including immunopathogenesis, pharmacology, and MOAs [3, 4, 7, 12, 13]. Flow cytometry profiling of TILs, as well as blood and tissue immune cell analysis, is the most used methodology for immunophenotyping [3–5, 7], while RNAseq and microarray [8–10] methods are the choice of methods to examine immuno-genomics of mouse tumors [3–5, 7]. The present report describes a new IO-targeting NGS panel for mouse immuno-genomics analysis, and discusses the potential applications and advantages, particularly in the context of the commonly used RNAseq and microarray assays.

## Materials and methods

### *In vivo* syngeneic mouse studies

Syngeneic mouse tumor models (MC38 colon cancer and Hepa1-6 liver cancer in C57BL/6 mice), as subcutaneously transplanted tumors in ICI pharmacology investigations, have widely been described [3, 4, 7]. CD4^+^ T-cell depletion experiment using anti-mouse CD4 antibody was also previously described [3]. All the protocols and procedures involving the care and use of animals were reviewed and approved by the Crown Bioscience Institutional Animal Care and Use Committee (IACUC) prior to conducting the studies (animal use protocol number AN-2004-12), in accordance with AAALAC (Association for Assessment and Accreditation of Laboratory Animal Care) guidelines as reported in the Guide for the Care and Use of Laboratory Animals, National Research Council (2011). All animal experimental procedures were under sterile conditions at SPF (specific pathogen-free) facilities and conducted in strict accordance with the Guide for the Care and Use of Laboratory Animals from the National Institute of Health, AVMA (2020) and ARRIVE guidelines [14]. Mice were supplied with irradiated standard rodent chow and 0.2μm filtered, autoclaved reverse osmosis water ad libitum. Mice were housed in groups of up to five mice per cage in polysulfone individually vented cages, with enrichment provided in each cage. Daily cage side observations were conducted and weekly clinical observations by experienced technical staff, which may require euthanasia. These include chronic and/or severe diarrhea leading to moderate to severe dehydration, severe anemia indicated by pale feet and ears, evidence of infection that is not readily treatable, inability/unwillingness to ambulate to reach food or water, blood discharge, labored breathing, emaciated or moribund condition. Mice showing a net body weight loss >20% compared to baseline weight measurement were euthanized. Mice were euthanized by CO_2_, followed by a secondary euthanasia method of cervical dislocation to alleviate suffering. Tumor growth inhibition, through twice weekly tumor volume measurement (1/2 length x width^2^, maximum size <2000mm^3^), was used as pharmacology readout.

### Flow cytometry analysis of TILs and immunogenomics analysis of bulk syngeneic tumors

Baseline (untreated) or treated tumors (ICI or anti-CD4 Antibody) were harvested at 500~700mm^3^ and were subjected to four different types of analysis. First, multi-color flow cytometry analysis was used for TIL analysis as described previously in detail [3, 5]. Second, the same tumor samples were subjected to RNAseq analysis as previously described [3, 15]. Third, microarray analysis, NanoString’s PanCancer Mouse IO 360 Panel [8, 10], of the same syngeneic tumors followed the same sample collection and subjected to the testing process per manufacturer recommended procedure.

Fourth, the same samples were subjected to NGSmIO panel test, as per experimental procedure as briefly below, total RNA was prepared from samples (*e*.*g*. tumors) using RNeasy Mini Kit (QIAGEN, Cat.74106) per manufacturer’s instruction. The quantity and quality of the total RNA are assessed using NanoDrop 2000 and Agilent 2100 BioAnalyzer. Only high-quality RNA sample (OD260/280 = 1.8–2.0, OD260/230≥2.0, concentration> 20ng/μL, amount>1μg, volume>30μL, RIN (RNA integrity number) ≥7) is used for the library construction and sequencing. PolyA mRNA was the isolated from the total RNA samples using two serial rounds of binding to oligo (dT) magnetic particles (Agilent) then chemically-fragmented to a size appropriate for RNA sequencing library preparation using RNA Seq Fragmentation Mix (Agilent). Using these short fragments as templates, first stranded cDNA was synthesized by RNA Seq First Strand Master Mix (Agilent) and Actinomycin D (Agilent), followed by purification using AMPure XP Beads (Beckman Coulter). Second-strand cDNA synthesis and end repair were then performed using RNA Seq Second Strand plus End Repair Enzyme Mix (and RNA Seq Second Strand + End Repair Oligo Mix, Agilent), followed by purification using AMPure XP Beads (Beckman Coulter). The purified cDNA was then subjected to cDNA 3’ ends dA-tailing using RNA Seq dA Tailing Master Mix (Agilent) and adaptor ligation using SureSelect Ligation Master Mix (Agilent), followed by purification using AMPure XP Beads (Beckman Coulter). The adapter-ligated cDNA library was then amplified by pre-capture PCR Reaction Mix (Agilent) and purification. After cDNA library construction, Qubit 3.0 fluorometer dsDNA HS Assay (Thermo Fisher Scientific) was used to quantify concentration, while the size distribution was analyzed using Agilent BioAnalyzer 2100 (Agilent). cDNA library with good quality and concentration was then used for the sequencing library hybridization using SureSelect Block Mix (Agilent) and RNA Capture Library Hybridization Mix (Agilent). Capture of hybrids was performed by streptavidin beads (prepared from Dynabeads M-270 Streptavidin Beads (Thermo Fisher Scientific)). Sequencing libraries was cleaned up after PCR amplification of captured libraries and indexing. After library quality control (QC), MGI Easy Universal Library Conversion Kit (MGI) with High-Throughput Pair-End Sequencing Primer Kit (MGI) was used to generate clusters. Paired-end sequencing will be performed using a MGISEQ2000 system following MGI-provided protocols for 2 x 150 paired-end sequencing.

### In silico bioinformatics analysis of immuno-genomic raw data

For gene expression profile preprocessing, raw sequencing data derived from NGSmIO panel were mapped to 1080 genes using pseudo aligner kallisto [16]. 1080 gene expression values were represented by log2 transformed Transcript per Million (TPM).

The quality of RNAseq fastq raw reads was checked by FastQC software (Babraham Bioinformatics, https://www.bioinformatics.babraham.ac.uk/projects/fastqc/). The adapter and low-quality sequences were trimmed by Trimmomatic software [17]. The reads were mapped to reference genes (ENSEMBL GRCh37.66) by Bowtie [18] software, and gene expression was calculated by RSEM [19] software. The final expression values are log2-transformed TPM values. Microarray raw intensity counts of 770 genes are quality controlled and normalized by R package nanostring [20].

For cross-platform analysis, the global clustering was performed by principal component analysis bases on all gene expression from NGSmIO panel and microarray. Expression matrices of 746 shared genes are retained to perform pairwise Spearman correlation analysis for related samples from NGSmIO panel, microarray and RNAseq. Pathway and immune signature scores were computed by gene set variation analysis [21] on NGSmIO panel and RNAseq based on defined signature gene sets within the NGSmIO panel, and immune signature scores were correlated with TIL numbers per mg tumor derived flow cytometry. Immune cell proportions in NGSmIO panel and RNAseq data are predicted by deconvolution analysis using EPIC [22] and were correlated with TIL fractions in total cells derived by flow cytometry. A schematic workflow is shown in S1A Fig.

## Results and discussion

### The design and construction of mouse IO NGS panel

**Fig 1:**
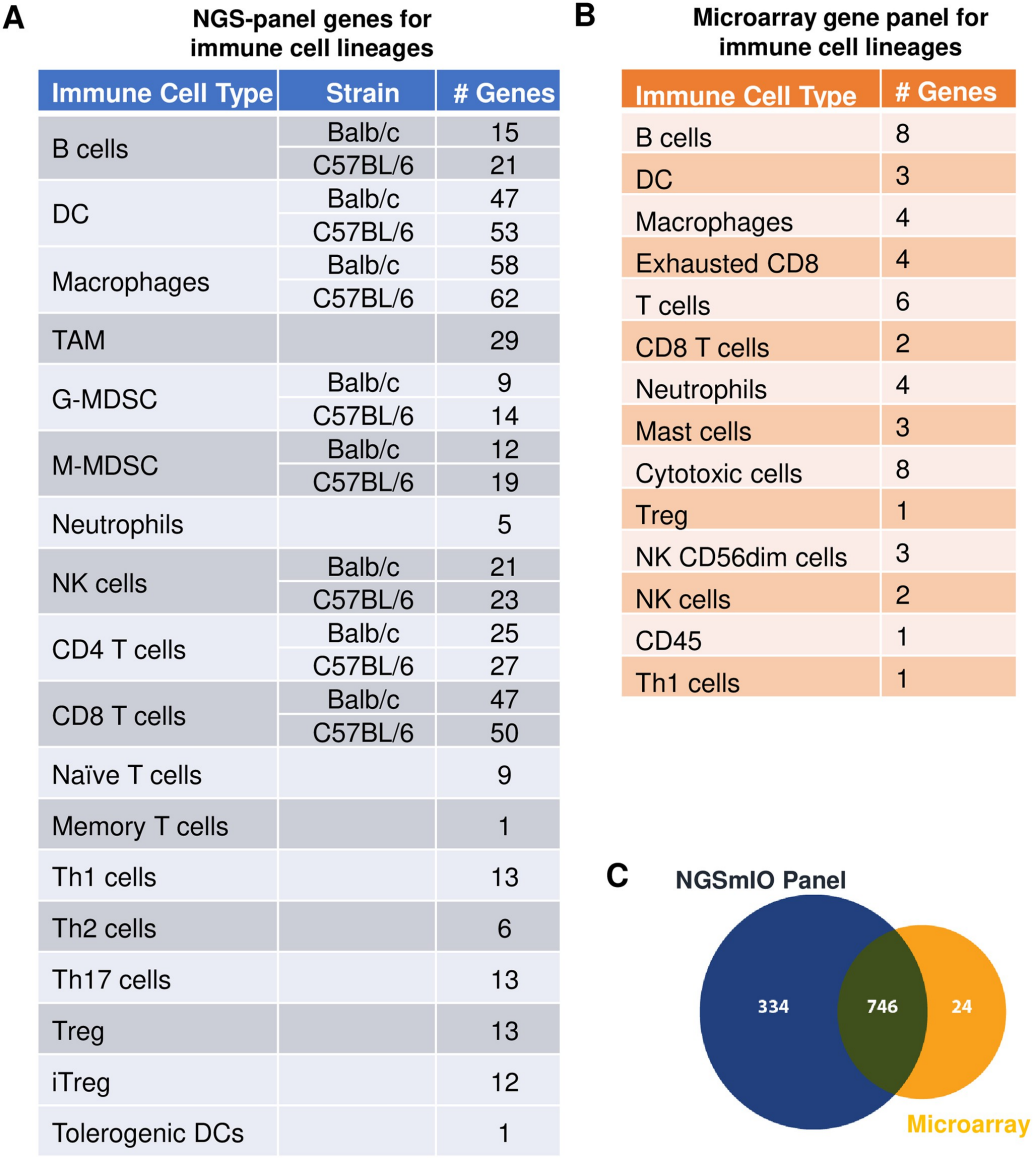
IO gene panel design summarizing microarray versus NGSmIO gene coverage. Number of signature genes for immune cell lineages in A. NGSmIO panel and B microarray gene panel. C. Venn diagram of common and unique genes included in NGSmIO panel (blue).and microarray panel (yellow).

With the objective of constructing a mouse IO NGS panel for measuring transcripts of immunology/IO interests, we selected 1080 genes, representative of various immune cell lineages (Fig 1A) and immunological processes (S1B Fig), to be included in the panel. The detailed list of 1080 IO genes and the relevance is shown in S1 Table. The panel covers 11914 coding exons regions, with each region including 10 base extension from both 3’ and 5’ ends of regions, and the total region size of 2.210Mbp.

The gene coverages of NGSmIO panel was compared with the widely used mouse IO panel based on microarray technology (NanoString’s PanCancer Mouse IO 360 Panel) [8–10], with 770 genes listed in S2 Table), and immune cell lineages represented by these genes in Fig 1B. There were 746 genes that overlapped between the two IO panels, with the NGSmIO panel including significantly more IO genes (334 unique genes, as opposed to 24 unique genes for microarray panel), particularly for the unique-genes representative of various lineage of immune cells (Fig 1C). The NGSmIO panel also added additional reference genes to ensure the precision measurement of gene expression. Overall, the additional genes in NGS panel were designed to be more comprehensive in covering IO related genes and cell lineages, and more precise in quantification.

### Correlation between NGSmIO panel and microarray panel

**Fig 2:**
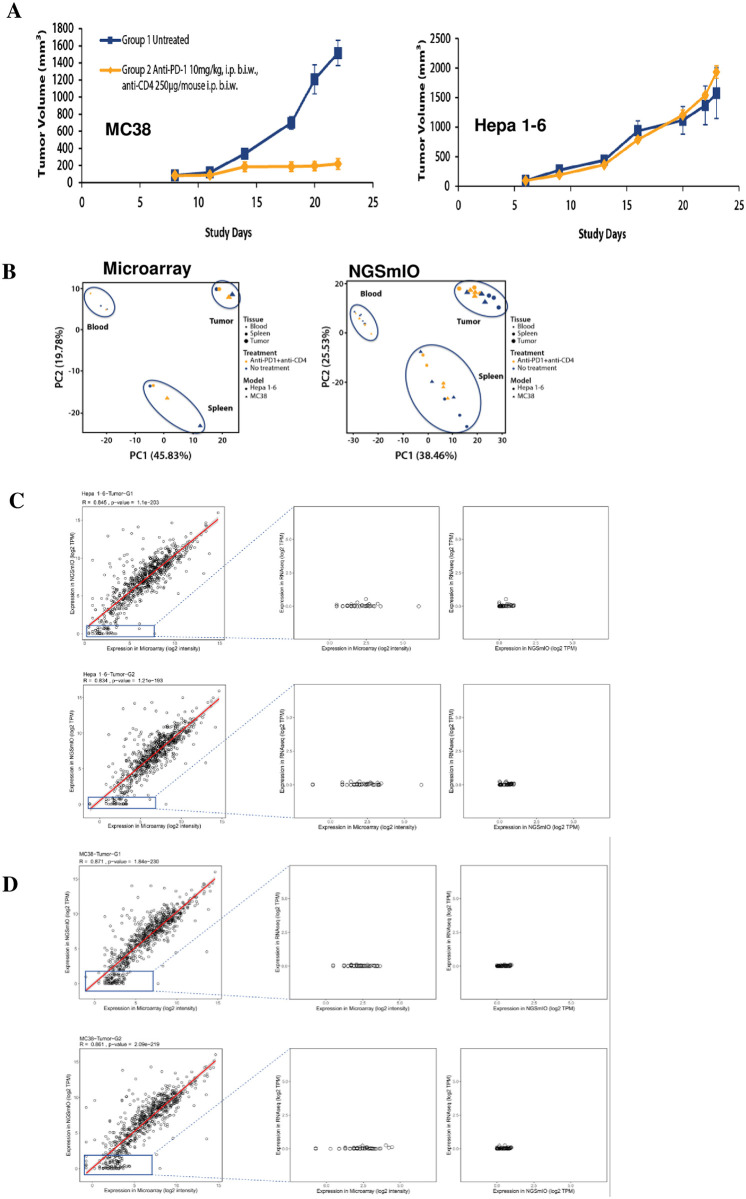
Comparison of NGSmIO and microarray platforms by testing samples collected from syngeneic tumor pharmacology study. A. Tumor growth curves for *in vivo* study with mice bearing either MC38 or Hepa1-6 subcutaneous tumors which were treated with 10μg/kg anti-PD-1 plus 250mg/mouse i.p. biweekly (Group 2, orange ♦, n = 3/group) or untreated (Group 1, blue ■, n = 3/group). Samples of tumor, spleen, and blood were collected from all animals on termination day (n = 3/group); B. Global clustering using Principal Component Analysis (PCA) for samples collected from microarray versus NGSmIO panels. Left: Microarray panel, n = 1/group/tissue; Right: NGS panel, n = 3/group/tissue; High Dynamic Range of NGSmIO Panel Enables Sensitive Detection of Low Expressing Genes in C) Hepa1-6 or D) MC38.

To test the NGSmIO panel in a real experimental setting, we compared the output to two previously described experiments [3]: 1) syngeneic MC38 tumors without treatment (G1) and ones treated by anti-CD4 and anti-PD-1 monoclonal antibodies together (G2); 2) Hepa1-6 without treatment and treated with the same combo-treatment. The tumor responses are shown in Fig 2A, where depletion of CD4+ T cells by anti-CD4 antibody combined with anti-PD-1 antibody resulted in retardation of MC38 tumor growth, but in contrast no effect was seen in Hepa 1–6 tumors (Fig 2A). The samples from tumors, bloods and spleens were then collected and processed by either NGSmIO panel or microarray analysis. Principal Component analysis (PCA) was first performed which displayed separated clusters by tissues type for both assays (Fig 2B). We also noticed intra-group variance exists among the samples. However, as expected, NGSmIO-panel data is clustered similarly to that of microarray in general, suggesting it can be used as an effective way to profile IO transcripts.

Correlation analysis between NGSmIO and microarray panels on shared genes revealed a high correlation (Spearman correlation coefficients R>0.83) across 12 paired samples (Table 1 and Fig 2C Hepa 1–6 tumors and Fig 2D MC38 tumors)), supporting the notion that both panels measure gene expression in a comparable way. However, there was notable difference in the lower expression range, where genes displaying little or no expression in the NGSmIO panel while being displayed with varying degree of expression in the microarray method in both tumor tumors (Fig 2C Hepa 1–6 and 2D MC38, left panel). When comparing these low expression genes against whole transcriptomic data (RNAseq), it seems significant high background noise in the microarray panel (middle panel, Fig 2C and 2D) as compared to the NGSmIO panel (right panel, Fig 2C and 2D) in these low expression range. In conclusion, the broader dynamic range of NGS panel enables more accurate detection of low expressing genes.

**Table 1:** Gene expression correlation between NGSmIO panel and microarray panel on the shared genes. Spearman correlation coefficient and correlation p-value for 12 paired samples detected from NGSmIO panel and microarray panel based on expression data of 746 shared genes are given.

Sample	Spearman correlation coefficient	p-value
Hepa 1-6-Blood-G1	0.867	9.94E-227
Hepa 1-6-Blood-G2	0.848	3.99E-206
Hepa 1-6-Spleen-G1	0.830	4.31E-190
Hepa 1-6-Spleen-G2	0.834	1.74E-193
Hepa 1-6-Tumor-G1	0.845	1.1E-203
Hepa 1-6-Tumor-G2	0.834	1.21E-193
MC38-Blood-G1	0.847	4.1E-205
MC38-Blood-G2	0.871	2.16E-231
MC38-Spleen-G1	0.860	3.23E-219
MC38-Spleen-G2	0.859	3.22E-217
MC38-Tumor-G1	0.871	1.84E-230
MC38-Tumor-G2	0.861	2.09E-219

### Correlation with RNAseq: NGSmIO-panel vs. microarray

**Fig 3:**
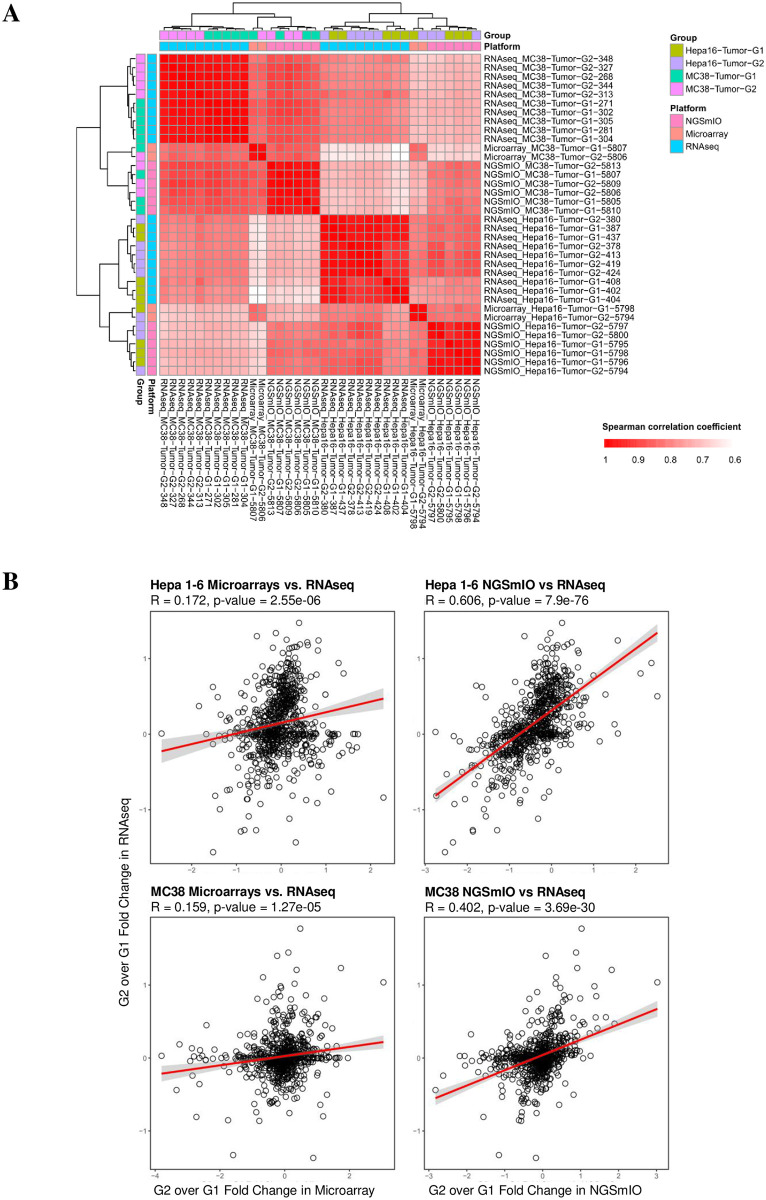
Comparing the NGSmIO and microarray against RNAseq. A) Clustering tumor sample RNAseq data with those of the same two IO panel (NGS vs. microarray) respectively with RNA-Seq Data in tumor samples; B) correlation (linearity) in fold changes between the treatment groups of NGSmIO and microarray against RNAseq.

We next computed the pairwise Spearman correlation between tumor samples detected by RNA-seq, NGSmIO-panel and microarray using the expression of 746 shared genes, and then clustered these samples from different platforms based on their correlations. The results demonstrated that global gene expressions of both assays are clustered together with RNA-seq data under the same pharmacological conditions (groups) (Fig 3A). However, NGSmIO panel has better correlation with RNA-seq than microarray (Fig 3A) for both tumor models.

When the expression changes in treatment over vehicle groups are compared between the two IO panels and RNA-seq, the NGSmIO panel demonstrated significant better correlation with RNAseq than the microarray panel (Fig 3B), where NGSmIO panel can accurately detect gene expression changes under treatment but microarray cannot. Specifically, in comparison of expression changes for 746 genes between Hepa 1–6 tumors under anti-CD4 (a depletion antibody) and anti-PD-1 combo-treatment conditions (G2) and no treatment condition (G1), the overall Spearman correlation coefficient for expression fold changes detected by RNAseq and NGSmIO panel is 0.604 (p-value = 7.9E-76) while the overall Spearman correlation coefficient for expression fold changes detected by RNAseq and microarray panel was only 0.172 (p-value = 2.55E-6, poorer concordance). Similar observation existed for expression changes for MC38 tumors detected by the NGSmIO panel and RNAseq (R = 0.402, p-value = 3.69E-30), as compared to those detected by microarray panel and RNAseq (R = 0.159, p-value = 1.27E-5) (Fig 3B).

### NGSmIO panel detection is 10 times more sensitive as compared to RNAseq with a defined amount of total sequencing data

One of the objectives to create the NGSmIO panel was to focus on the immunological and IO related genes, by excluding most other unrelated genes, so to enable more in-depth and sensitive analysis on the gene collection of interests. We next set out to evaluate the sensitivity of the NGSmIO panel by comparing it with the whole-transcriptome sequencing (RNAseq) at the same data amount. We performed both sequencing methods on 209 murine tumor samples derived from 21 syngeneic models (S3 Table), and compared the reads aligned to 1080 shared genes, *i*.*e*., 1080 IO core genes included in the NGSmIO panel. The average read counts detected by the NGSmIO panel for 1080 genes across 209 pairs of tumor samples is 2863.32GB, which is on average 12.38±2.10 times of that by RNAseq (254.64GB) (S3A Table). Particularly in hematological malignances such as J558 myeloma, and lymphomas including A20, EG7-OVA and EL4, the normalized read counts ratios between the NGSmIO panel and RNAseq reached 18.25±0.61 and 14.95±1.08, respectively, while in other non-immune cell driven tumors the mean ratio is 11.57±1.17 (S3B Table). Overall, these results revealed that the NGSmIO panel has at least 10-fold higher sequencing depth on target IO genes than the conventional RNAseq on the basis of the same data quantity, and suggest that IO panel will be more sensitive in the detection and quantification of IO genes, particularly for the low expressed genes, many of which are in the tumors (*e*.*g*. TILs).

### Detection of pharmacodynamic change of interferon-γ (IFN-γ) expression levels upon anti-PD-1 and anti-CD4 combination treatments in MC38 and Hepa 1–6 tumors

**Fig 4:**
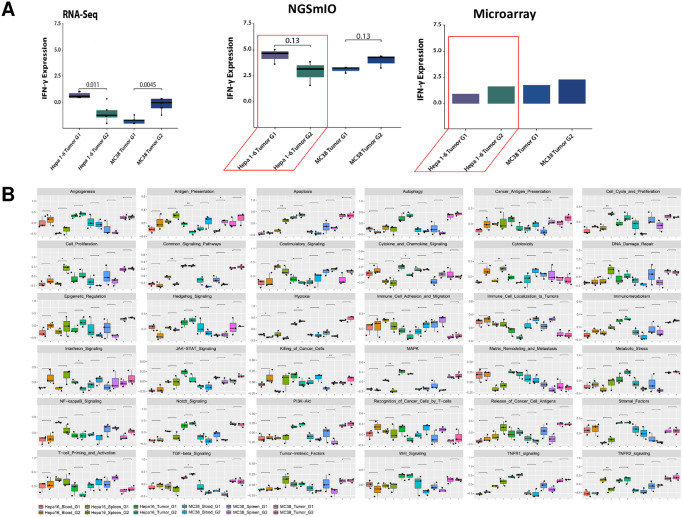
Pathway analysis identify mechanism of action of immunotherapy. A. Changes in IFN-γ expression levels during treatment, as detected by different methods: RNAseq, NGSmIO panel and microarray panel. B. Gene set variation analysis on 36 IO related pathways across tissues (blood, spleen and tumor) and treatment groups (Group 1, untreated and Group 2, treated with 10mg/kg anti-PD-1 plus 250μg/mouse i.p. biweekly) in MC38 and Hepa 1–6.

We next set out to examine a specific gene expression change upon combination treatment with anti-PD-1 and anti-CD4 antibodies in MC38 and Hepa1-6 tumors as described previously [3]. As shown in Fig 4A, the NGSmIO panel detected an increase in IFN-***γ*** expression in MC38 tumors, in contrast to the decrease in Hepa1-6, both similarly observed by RNAseq and proteomics analysis [3]. However, the increase was less evident using the microarray method in MC38 tumor and actually, opposite observation (increase) was seen in Hepa1-6 (Fig 4A). Furthermore, by Gene Set Variation Analysis (GSVA) [21] on 36 IO pathways, we discovered that, in contrast to MC38, antigen presentation was significantly depressed (p-value < 0.01) as well as interferon, chemokine and cytokine signaling (p-value < 0.05) by anti-PD-1 treatment and CD4 depletion in Hepa1-6 tumors, whereas hypoxia and TGF-β signaling were significantly induced (p-value < 0.05) (Fig 4B). Taken together, the NGSmIO panel was more accurate than microarray to detect the pharmacodynamic changes for MOA evaluation.

### Benchmarking the baseline TILs in 14 syngeneic tumors using the NGSmIO panel

**Fig 5:**
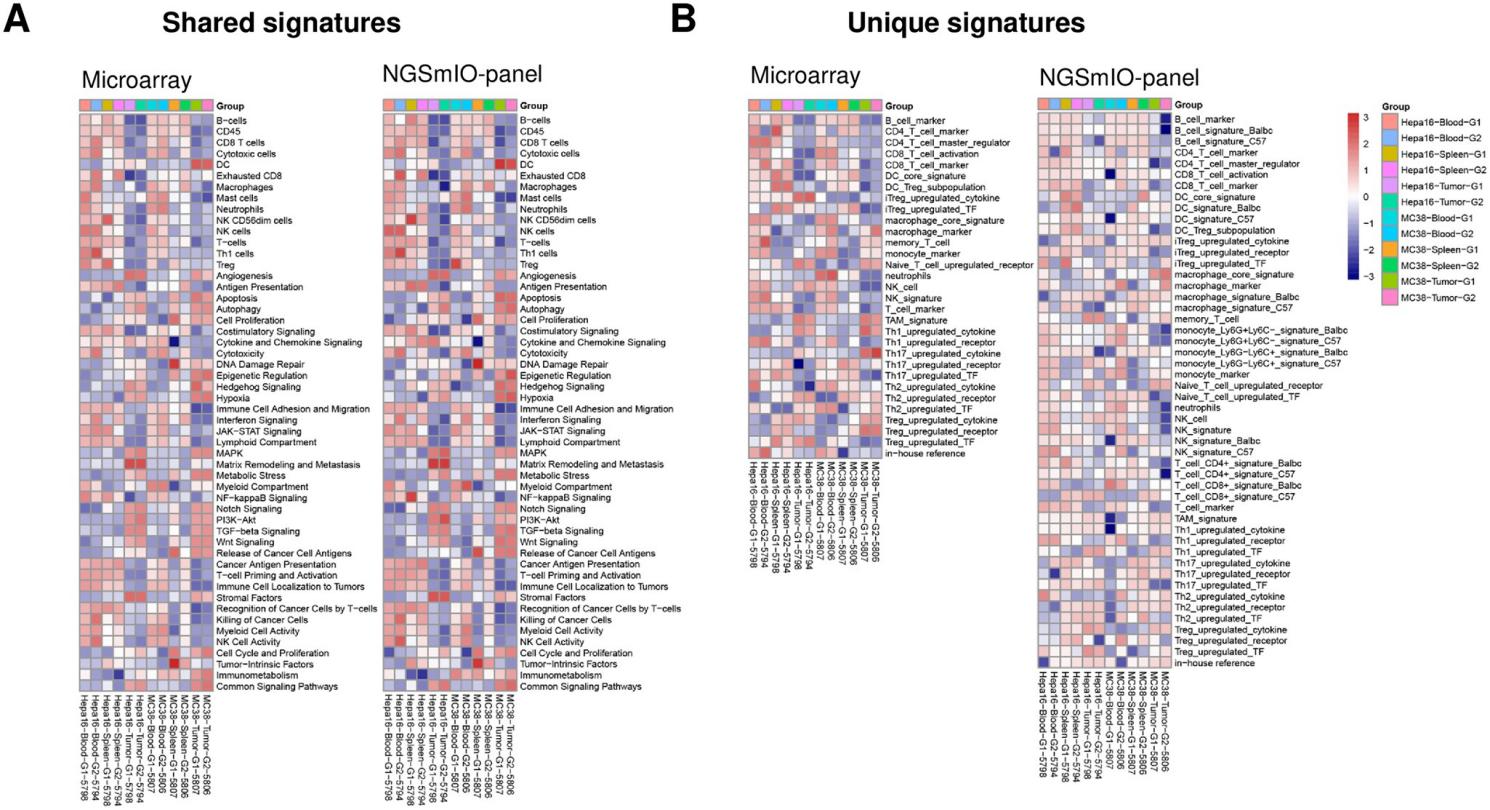
Gene signatures of immune cell lineages. NGSmIO panel vs. microarray panel. A. Similar expression patterns in shared signatures; B. NGS panel includes more depth immune cell signatures.

TIL analysis measures important tumor immunity parameters most commonly achieved by flow cytometry methods [3, 5], as well as other multi-parametric methods such as immunochemistry (IHC) and immunofluorescence (IF). One of the important features of the NGSmIO panel is that it includes lineage gene signature (Table 2) by design for detecting and measuring the rare TIL lineages. It is more sensitive than RNAseq as described above, while the available microarray does not include as comprehensive lineage-specific genes (Fig 5) and has limited accuracy in detection dynamic ranges as described above. We then set out to perform the detection and measurement of TIL lineages in a 14 syngeneic mouse tumor panel (10 mice per model) using transcript levels of the lineage corresponding genes, *e*.*g*., CD4 transcript representing CD4^+^ T-cells and CD8 transcript representing CD8^+^ T-cells, *etc*., as shown in Table 2 (S2 Fig). The estimated contents (% of TIL) of each lineage, based on the reads of the corresponding marker transcripts, are compared to the TIL determination of the same tumor models by flow cytometry using specific markers or marker combinations (“flow panel”) as previously described (5). For comparison, the same analysis was also performed using RNAseq data. The overall absolute cell numbers per mg tumor detected by FACS for 8 TIL populations, *i*.*e*., CD45^+^ total leukocytes, CD3^+^-/CD4^+^/CD8^+^ T cells, T_reg_, macrophages, monocytes and NK cells, were highly correlated with the NGSmIO panel and RNAseq detected signature scores defined for the corresponding cell populations, meanwhile NGSmIO panel showed a slightly better correlation than RNAseq (Table 2). This result suggests that the NGSmIO panel can be used to estimate the relative TIL components for both contents and relative ratios, similar to flow cytometry, and if standard curves can be created based on flow cytometry data, the NGS-panel can in theory provide % cell as flow cytometry.

**Table 2:** Correlation analysis of NGSmIO and RNAseq for 8 immune lineage signature scores and FACS derived cell count. Spearman correlation coefficient (Rho) and correlation p-value for 8 immune lineages by comparing NGSmIO-derived signature scores and FACS-derived cell counts, and RNAseq-derived signature scores and FACS-derived cell counts are shown. The signature genes used by NGSmIO and RNAseq, with markers used in FACS analysis, are provided in the last two columns.

Lineages	Rho (NGSmIO-FACS)	p-value (NGSmIO-FACS)	Rho (RNAseq-FACS)	p-value (RNAseq-FACS)	Markers used in NGSmIO and RNAseq	Markers used in FACS
CD45+ cells	0.597	7.06E-15	0.601	4.10E-15	Ptprc	CD45
T cells	0.556	< 2.20E-16	0.609	< 2.20E-16	Sh2d1a,Cd247,Cd3d,Cd3e,Cd3g,Il2ra	CD3
CD4+ T cells	0.375	5.01E-06	0.319	1.20E-04	Cd4	CD4
CD8+ T cells	0.642	1.18E-17	0.628	1.03E-16	Cd8a	CD8
Tregs	0.454	2.50E-08	0.343	3.72E-05	Cxcr1,Il15ra,Irak2,Nectin1,Tnfrsf11a,Traf2	FoxP3
Macrophages	0.637	< 2.20E-16	0.584	< 2.20E-16	Cd14,Fcgr3,Cd163,Cd68,Cd84	CD11b+ F4/80+Gr-1-
Monocytes	0.532	1.37E-11	0.461	9.85E-09	Itgam	CD11b+ F4/80-Gr-1med
NK cells	0.668	< 2.20E-16	0.760	< 2.20E-16	Eomes,Gzma,Khdc1a,Klrb1a,Klrb1b,Klrb1c,Klre1,Klrg1,Klrk1,Ncr1,Prf1,S1pr5	CD3-CD335+

## Conclusion

Mouse tumor models, such as syngeneic cell derived tumors or homograft tumors (4, 5), are important tools to investigate immunopathogenesis and immunotherapy of cancers, including MOA and proof of concept (POC). Multiparametric analysis of these tumors, including baseline and pharmacodynamic changes, are believed to be critical insights into aspects of tumor immunology, both tumor cells and TME. Multi-omics of different platforms, particularly those measuring gene expression, are proven to be robust and cost-effective. Among them, RNAseq, although an effective discovery tool, may not be as robust, cost-effective and productive as a specially tailored NGS panel in routine and standard characterizations of tumor immunity, for ease of analysis, sensitivity and high-throughput. On the other hand, NGS panel seems also have high detection/quantitation dynamic range than the widely used microarray platform. In particular, at the age of NGS technology becoming ubiquitous, an NGS panel can be particularly readily adopted for laboratory research for efficiency and lower costs. In particular, NGS panel is already widely used in the clinics as well as companion diagnostics (CDx) for the reasons mentioned here. It is also plausible that a preclinical NGS panel could have better translatability.

As a comprehensive IO panel, the NGSmIO can reveal disease pathway mechanisms and IO drug MOAs via mRNA-pharmacodynamic studies as we demonstrated in this report. In particular, as specifically designed, the lineage analysis of TILs, as an alternative to flow cytometry, could provide additional sampling advantages, without requirement of fresh tumor cells and facilitating the development of clinical applications. Furthermore, benchmarking the baseline of large panel of mouse tumor using the NGSmIO-panel together with pharmacology could also help to reveal the predictive biomarker of IO therapies (manuscript in preparation). Ultimately this panel could become a powerful tool in the laboratory for IO research.

## Supporting information

S1 Fig Overview of workflow and mouse I/O RNAseq composition. A. Schematic workflow of mouse I/O NGS panel process; B. Compositions of mouse I/O RNA-Seq Panel. Left panel: genes representing various immunological factors included in the NGS panel (total 1080 mouse transcripts); Right panel: genes representing various immune cell lineages and types.
(PDF)

S2 Fig Comparison of FACS analysis and signature scores. Scatter plots for 8 immune cell types measured by FACS analysis (cell number per mg) compared to signature score by mIO NGS panel (top) and RNAseq (bottom).
(PDF)

S1 Table Ensembl ID and gene symbol. List for 1080 genes in NGSmIO Panel.
(XLSX)

S2 Table NCBI accession number and gene symbol. List of all 770 genes in NanoString’s PanCancer Mouse IO 360 Panel.
(XLSX)

S3 Table Sequencing coverage comparisons on 1080 IO genes between NGSmIO Panel and RNAseq across 21 syngeneic models. **A.** Read count comparisons on 1080 IO genes for 209 tumors derived from 21 syngeneic models. **B.** Overall mean and standard deviation of read count fold change between NGSmIO Panel and RNAseq for specific categories of cancers. **C.** Cell line and mouse strain details for each model.
(XLSX)

S4 Table Data availability. Data description of raw NGS data in FASTQ format deposited to the Sequence Read Archive (BioProject accession number PRJNA878935).
(XLSX)
