# Supplementary material for: An NGS-based assay for accurate detection and quantification of immune gene expression in mouse tumor models
Source: PLoS One. 2024 May 20;19(5):e0303171. doi: 10.1371/journal.pone.0303171 (PMC11104603; doi:10.1371/journal.pone.0303171)
Supplement: S1 Fig — A. Schematic workflow of mouse I/O NGS panel process; B. Compositions of mouse I/O RNA-Seq Panel. Left panel: genes representing various immunological factors included in the NGS panel (total 1080 mouse transcripts); Right panel: genes representing various immune cell lineages and types. (PDF) [file pone.0303171.s001.pdf]

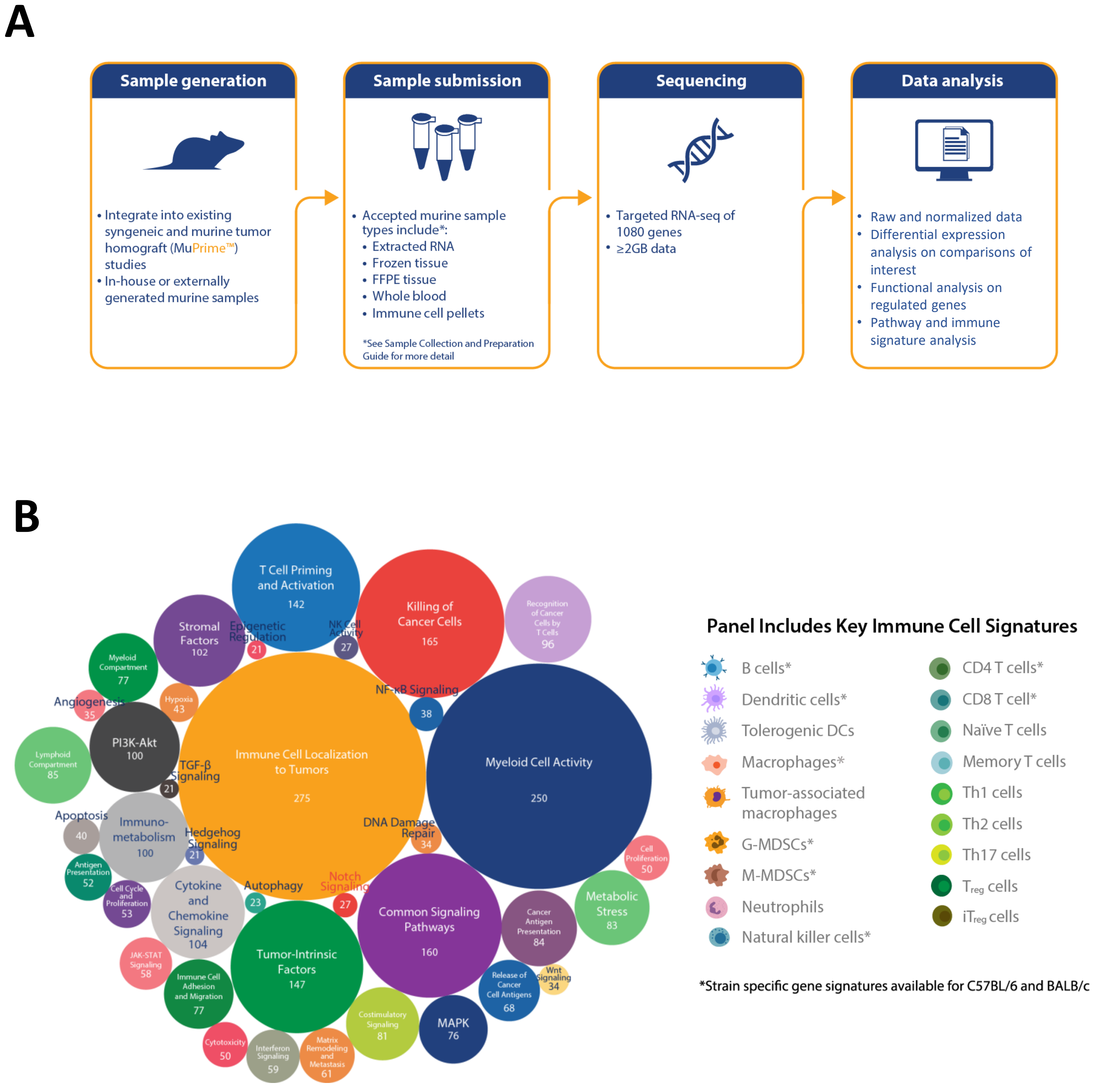

Supplement Figure S1
A. Schematic workflow of mouse I/O NGS panel process
B. Compositions of mouse I/O RNA-Seq Panel. Left panel: genes representing various immunological factors included in the NGS panel (total 1080 mouse transcripts); Right panel: genes representing various immune cell lineages and types.
