# Supplementary material for: An NGS-based assay for accurate detection and quantification of immune gene expression in mouse tumor models
Source: PLoS One. 2024 May 20;19(5):e0303171. doi: 10.1371/journal.pone.0303171 (PMC11104603; doi:10.1371/journal.pone.0303171)
Supplement: S2 Fig — Scatter plots for 8 immune cell types measured by FACS analysis (cell number per mg) compared to signature score by mIO NGS panel (top) and RNAseq (bottom). (PDF) [file pone.0303171.s002.pdf]

Supplementary Figure S2 Scatter plots for 8 immune cell types measured by FACs analysis (cell number per mg) compared to signature score by mIO NGS panel (top) and RNAseq (bottom)

CD45+

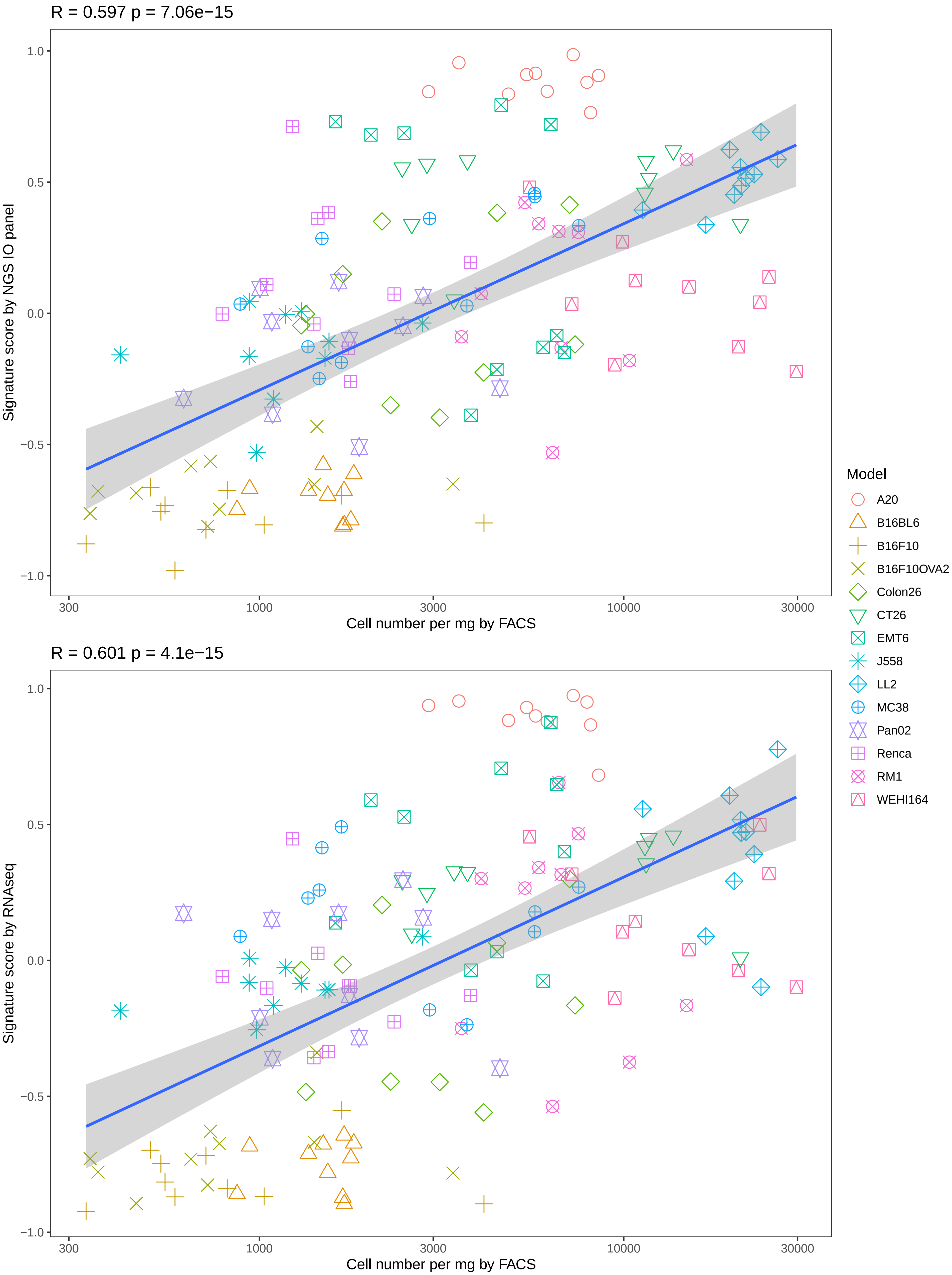

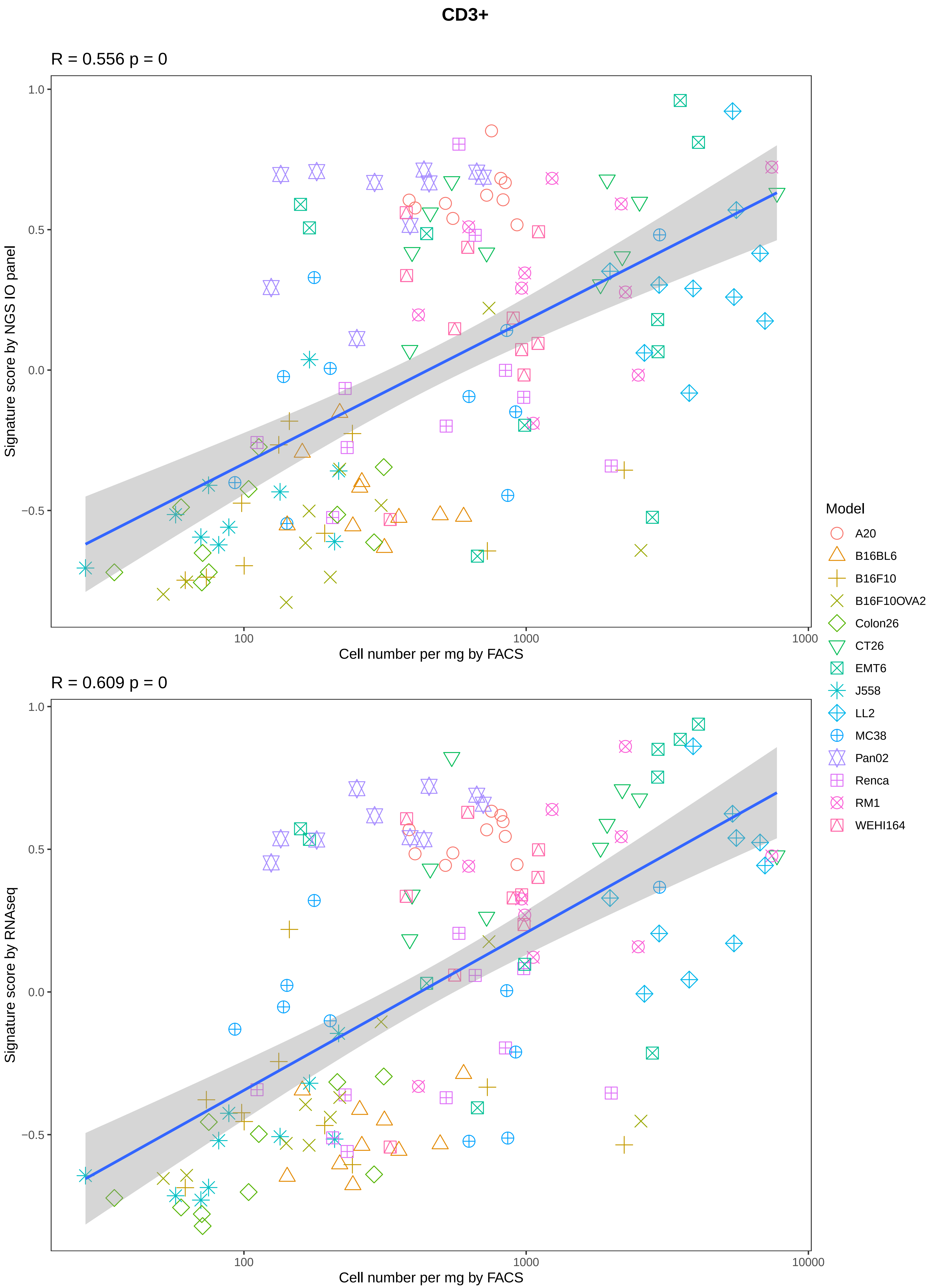
CD3+
R = 0.556 p = 0
1.0
0.5
0.0
−0.5
Signature score by NGS IO panel
100
1000
1000
Cell number per mg by FACS
R = 0.609 p = 0
1.0
0.5
0.0
−0.5
Signature score by RNAseq
100
1000
10000
Cell number per mg by FACS
Model
A20
B16BL6
B16F10
B16F10OVA2
Colon26
CT26
EMT6
J558
LL2
MC38
Pan02
Renca
RM1
WEHI164

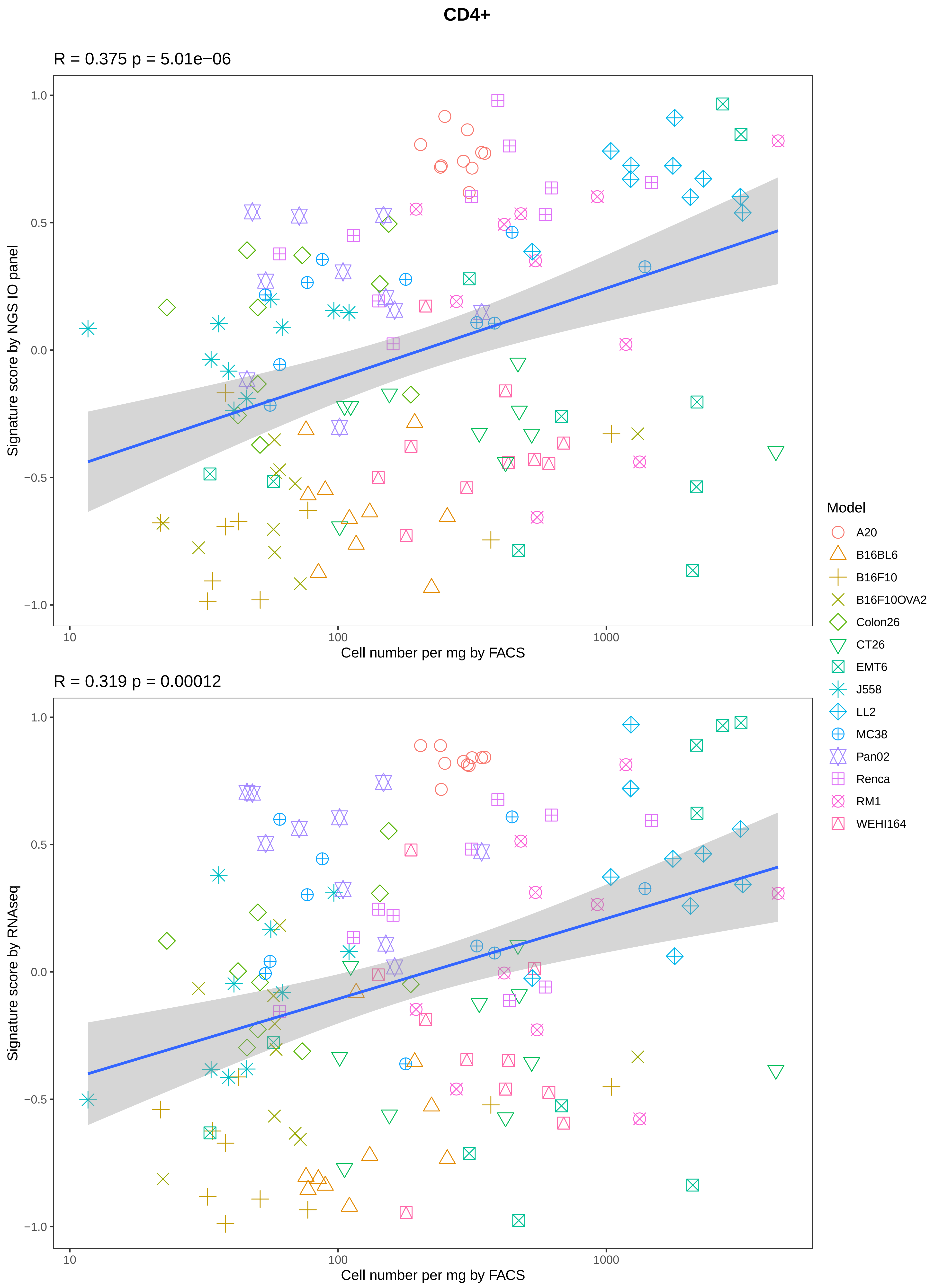
CD4+
R = 0.375 p = 5.01e−06
Signature score by NGS IO panel
Cell number per mg by FACS
R = 0.319 p = 0.00012
Signature score by RNAseq
Cell number per mg by FACS
1.0
0.5
0.0
−0.5
−1.0
10
100
1000
Model
A20
B16BL6
B16F10
B16F10OVA2
Colon26
CT26
EMT6
J558
LL2
MC38
Pan02
Renca
RM1
WEHI164

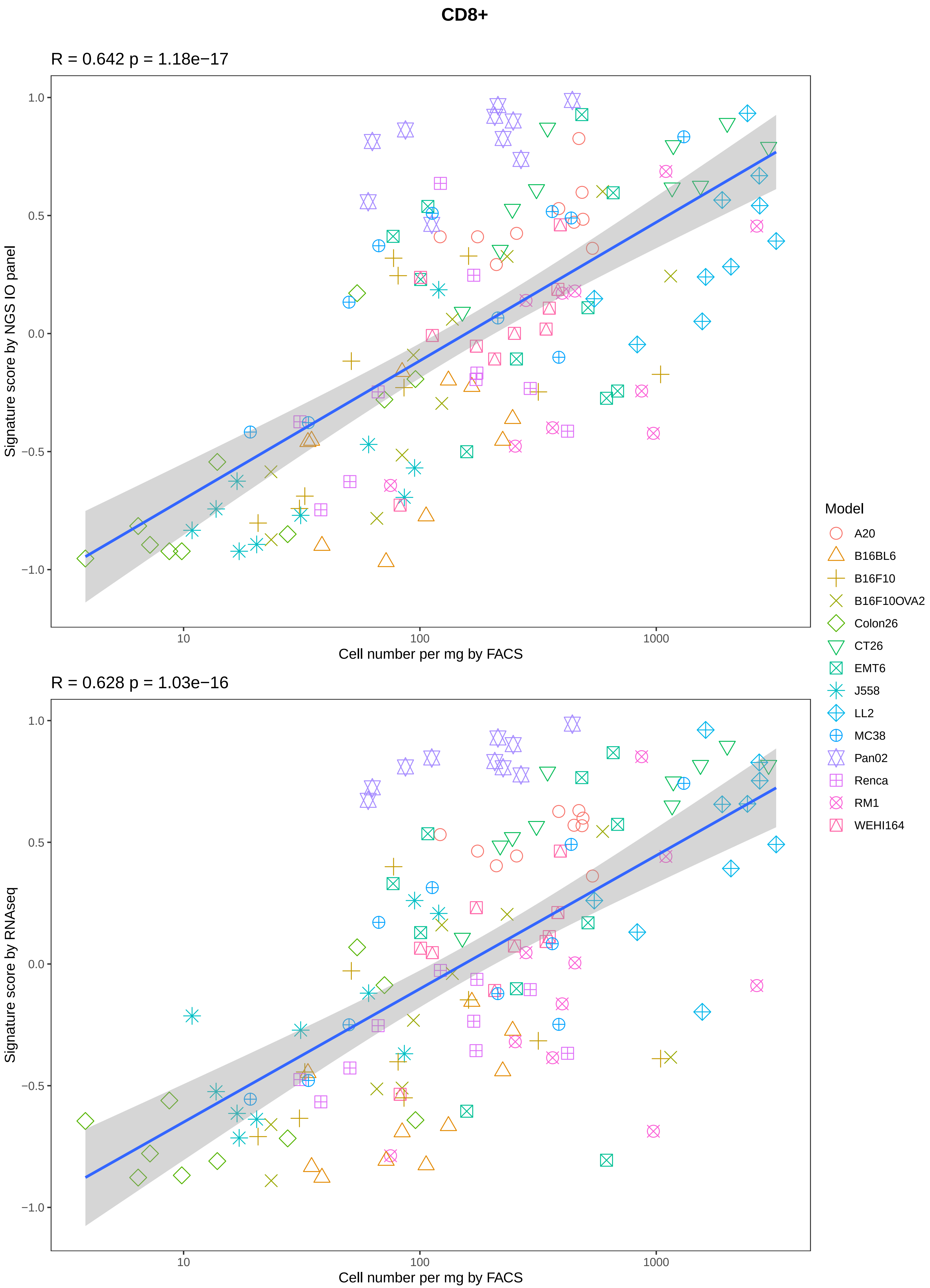

CD8+
R = 0.642 p = 1.18e−17
Signature score by NGS IO panel
Cell number per mg by FACS
R = 0.628 p = 1.03e−16
Signature score by RNAseq
Cell number per mg by FACS
1.0
0.5
0.0
−0.5
−1.0
10
100
1000
Model
A20
B16BL6
B16F10
B16F10OVA2
Colon26
CT26
EMT6
J558
LL2
MC38
Pan02
Renca
RM1
WEHI164

Tregs

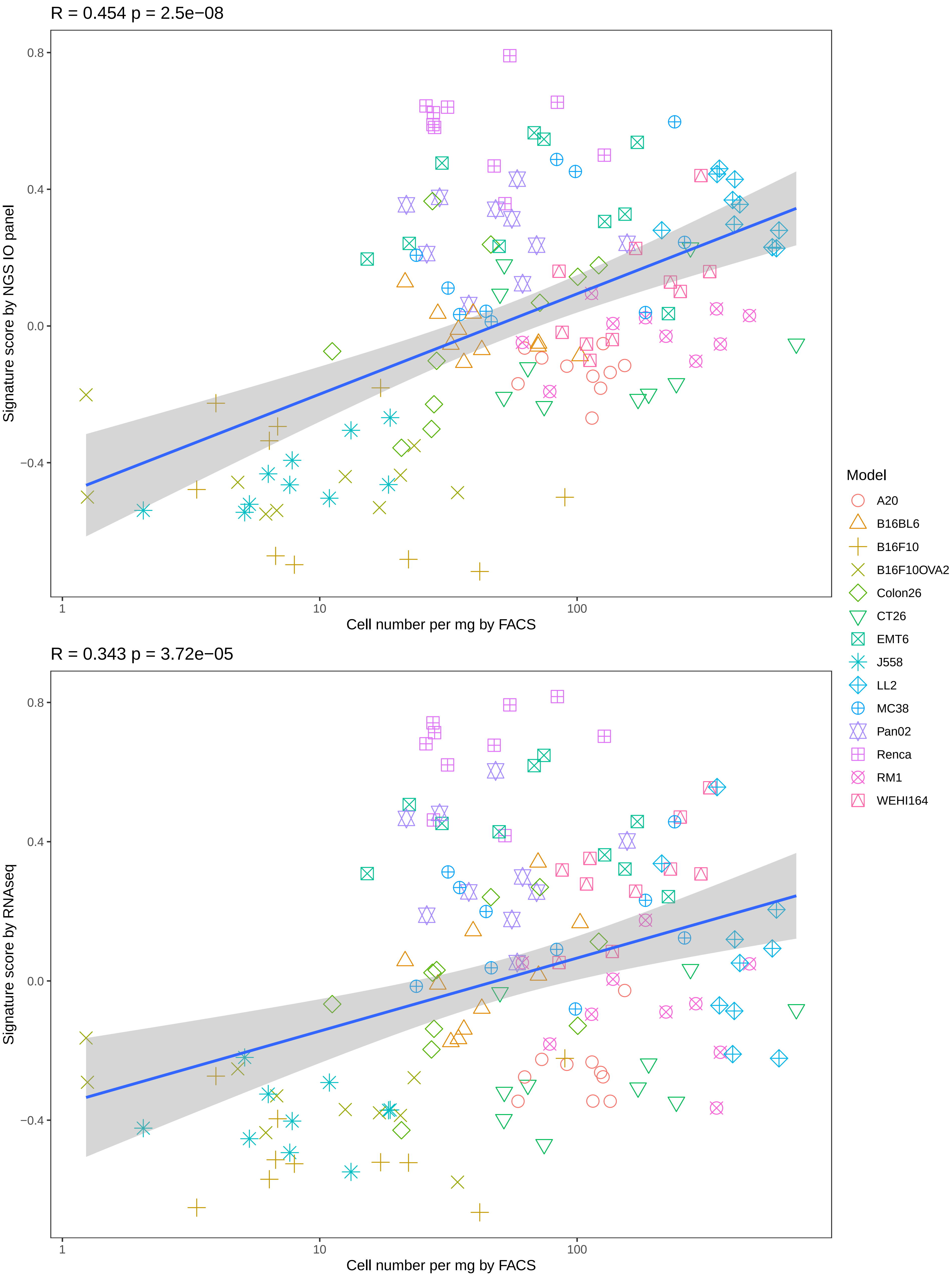
R = 0.454 p = 2.5e−08
Signature score by NGS IO panel
0.8
0.4
0.0
−0.4
1
10
100
Cell number per mg by FACS
R = 0.343 p = 3.72e−05
Signature score by RNAseq
0.8
0.4
0.0
−0.4
1
10
100
Cell number per mg by FACS
Model
A20
B16BL6
B16F10
B16F10OVA2
Colon26
CT26
EMT6
J558
LL2
MC38
Pan02
Renca
RM1
WEHI164

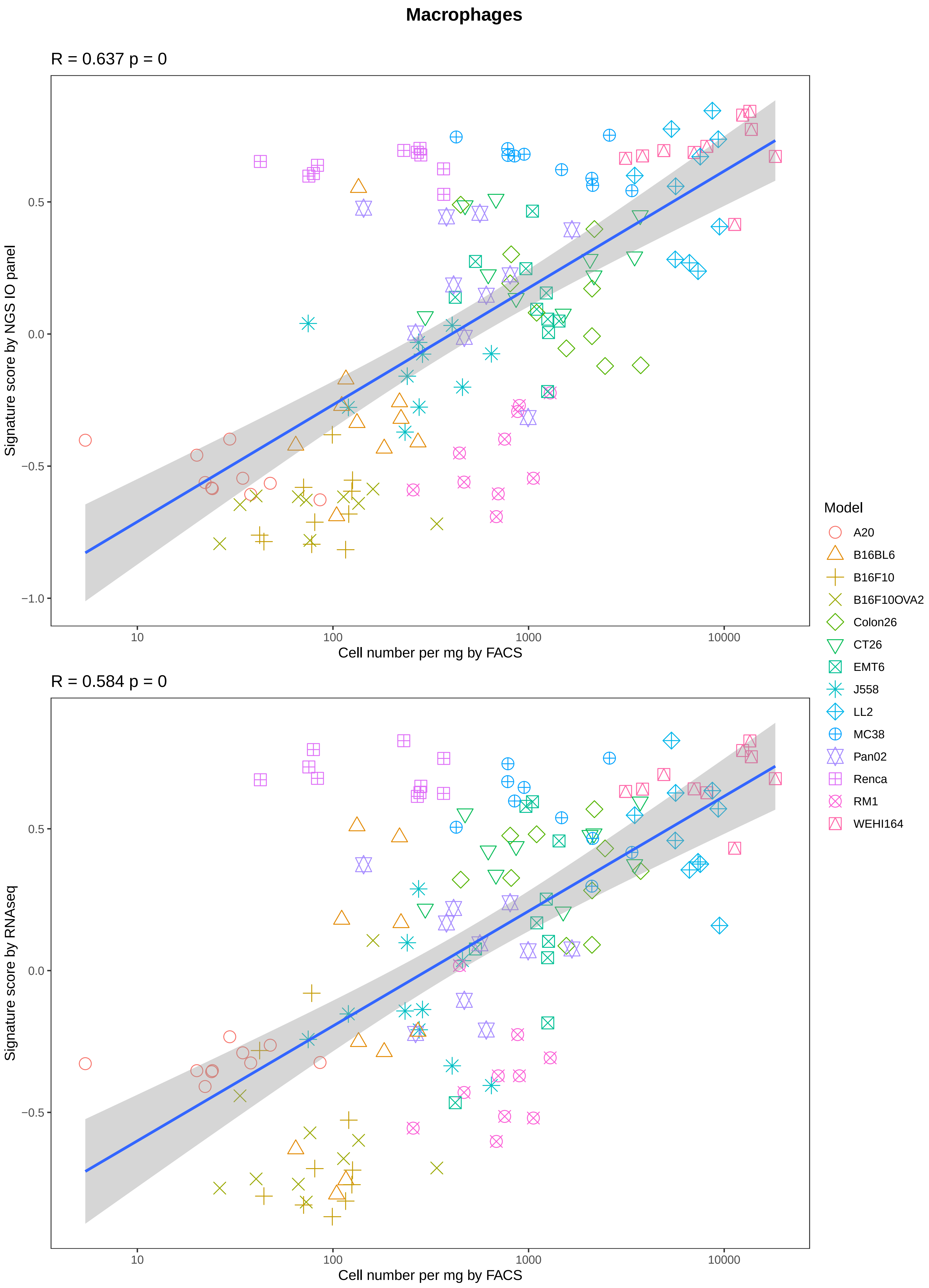
Macrophages
R = 0.637 p = 0
Signature score by NGS IO panel
0.5
0.0
−0.5
−1.0
10
100
1000
10000
Cell number per mg by FACS
R = 0.584 p = 0
Signature score by RNAseq
0.5
0.0
−0.5
10
100
1000
10000
Cell number per mg by FACS
Model
A20
B16BL6
B16F10
B16F10OVA2
Colon26
CT26
EMT6
J558
LL2
MC38
Pan02
Renca
RM1
WEHI164

Monocytes

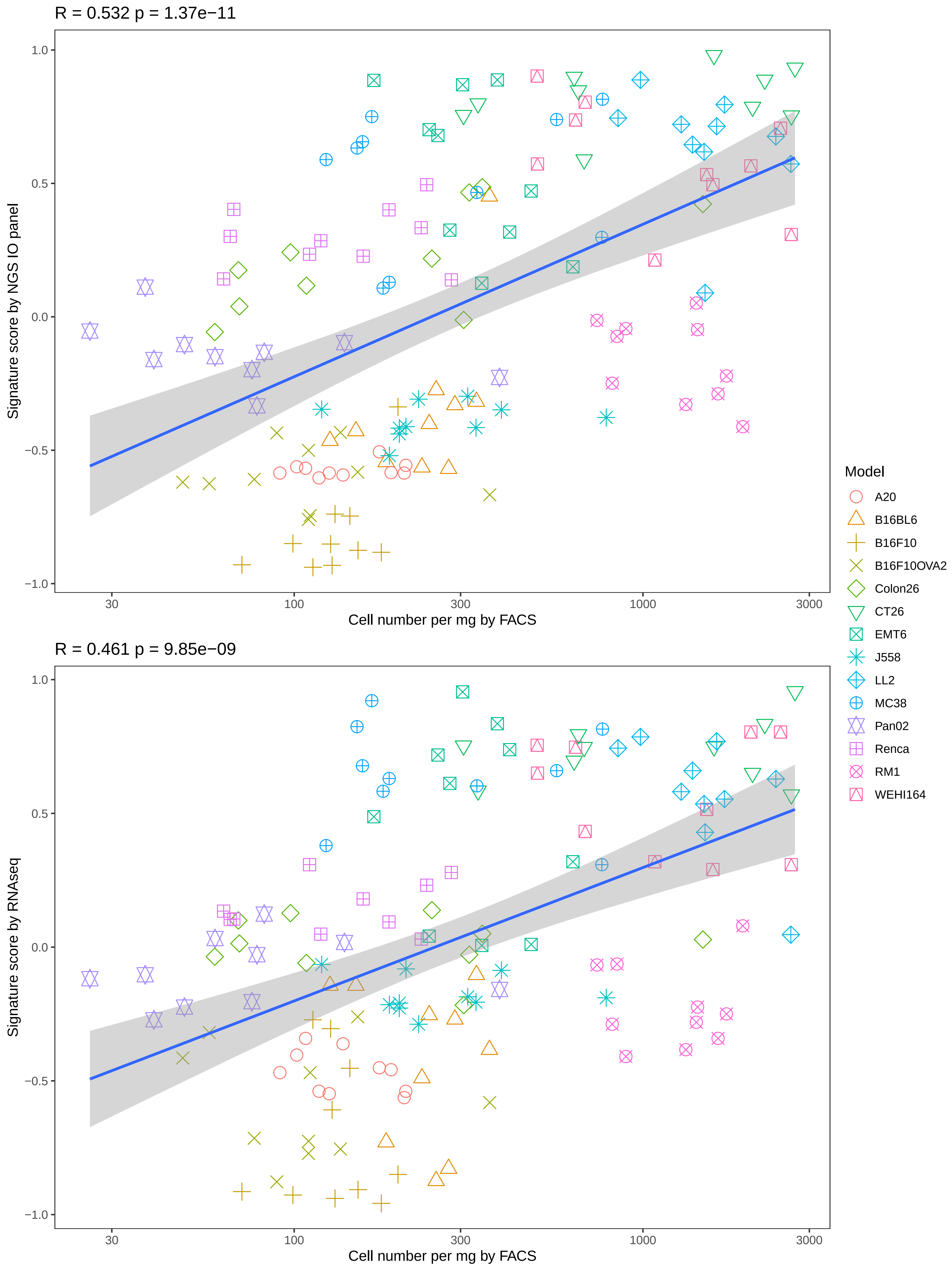

R = 0.532 p = 1.37e−11
Signature score by NGS IO panel
1.0
0.5
0.0
−0.5
−1.0
30
100
300
1000
3000
Cell number per mg by FACS
R = 0.461 p = 9.85e−09
Signature score by RNAseq
1.0
0.5
0.0
−0.5
−1.0
30
100
300
1000
3000
Cell number per mg by FACS
Model
A20
B16BL6
B16F10
B16F10OVA2
Colon26
CT26
EMT6
J558
LL2
MC38
Pan02
Renca
RM1
WEHI164

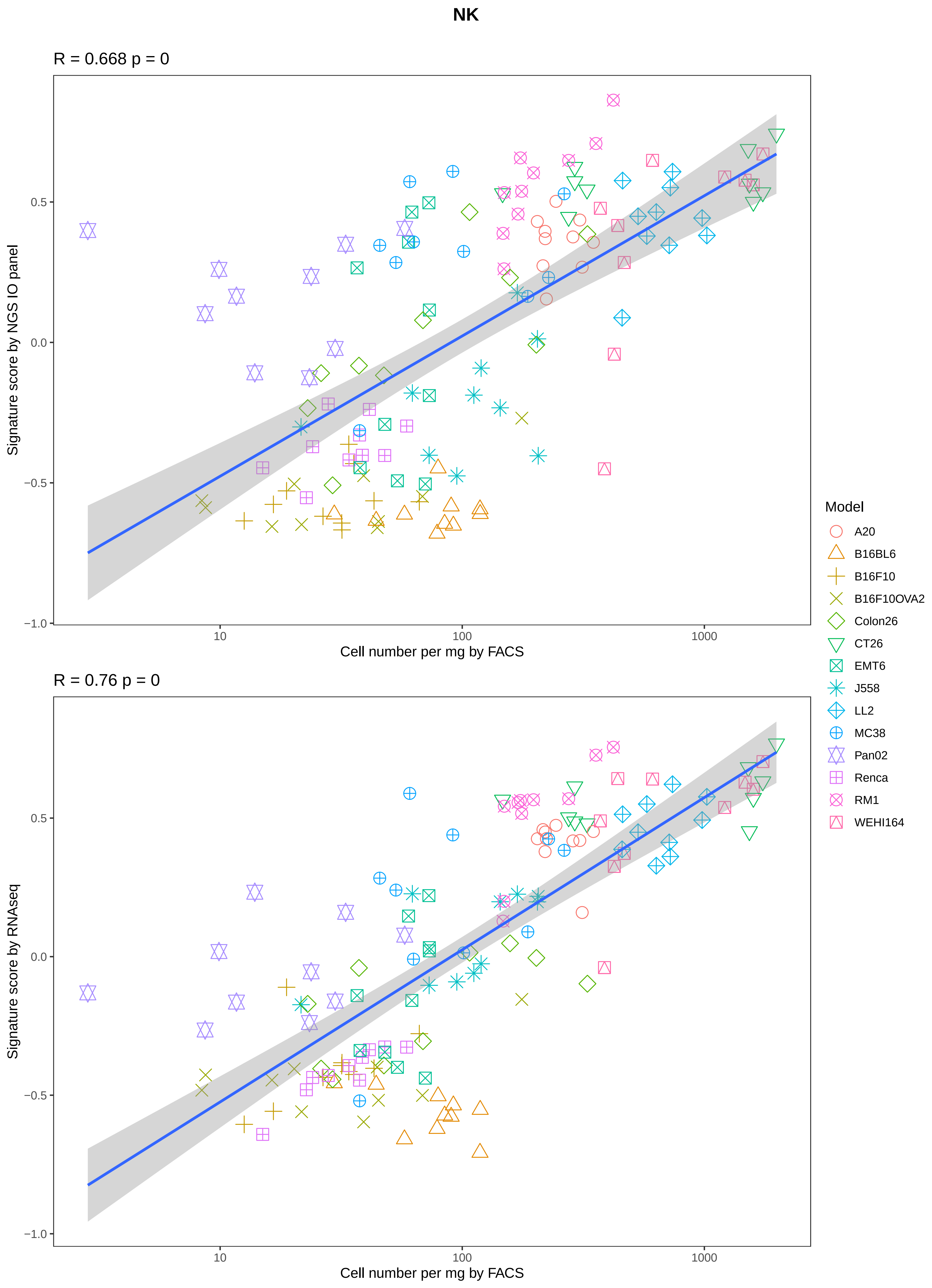
NK
R = 0.668 p = 0
Signature score by NGS IO panel
Cell number per mg by FACS
R = 0.76 p = 0
Signature score by RNAseq
Cell number per mg by FACS
0.5
0.0
−0.5
−1.0
10
100
1000
Model
A20
B16BL6
B16F10
B16F10OVA2
Colon26
CT26
EMT6
J558
LL2
MC38
Pan02
Renca
RM1
WEHI164
